# Music's context-dependent influence on oxytocin, social bonding, and emotion regulation: a systematic review

**DOI:** 10.3389/fcogn.2025.1678665

**Published:** 2026-01-02

**Authors:** Ting-Hsuan Chu, Chen-Gia Tsai

**Affiliations:** 1 Graduate Institute of Musicology, National Taiwan University, Taipei, Taiwan; 2 Graduate Institute of Brain and Mind Sciences, National Taiwan University, Taipei, Taiwan

**Keywords:** music therapy, oxytocin, context-dependence, social cognition, affect regulation, social behavior, ritual music

## Abstract

**Objective:**

This systematic review aims to explore how individual and group musical activities influence social bonding and emotion regulation through the oxytocinergic system.

**Methods:**

Following the PRISMA 2020 guidelines, a systematic search of PubMed, Embase, Scopus, Web of Science Core Collection, and PsycInfo was conducted to identify studies up to October 2024, supplemented by a manual search. One reviewer screened studies, extracted data, and assessed the study quality. Framework synthesis and narrative synthesis were conducted to integrate findings.

**Results:**

A total of 1,865 records were identified. After reviewing the full-text papers, 20 studies (seven randomized controlled trials and 13 quasi-experiments) were included, which involved 877 participants across healthy and clinical populations. The reviewed interventions included singing, playing instruments, listening to music, and music therapy. Most studies reported improvements in psychosocial outcomes, such as reduced anxiety and depression or enhanced social cognition, but they do not always align with peripheral oxytocin (OXT) changes. However, certain psychosocial outcomes or contexts revealed relatively consistent patterns in OXT responses, suggesting the presence of context-dependent modulation. Short-term interventions often reported detectable peripheral OXT changes, which only partially reflected the temporary activity of magnocellular OXT neurons in the hypothalamus. No significant changes in baseline peripheral OXT levels were observed after long-term interventions.

**Conclusion:**

Music-induced OXT responses are context-dependent. The bidirectional modulation of OXT supports social bonding and emotion regulation in musical contexts. Clinicians and music therapists should carefully consider therapeutic goals, individual differences, and environmental factors when designing music therapy.

## 1 Introduction

There is growing interest in music therapy, which has shown promise in promoting mental health and wellbeing (Bernatzky et al., 2011; Bhandarkar et al., 2024). Recent studies have begun to explore the underlying neural mechanisms of music's effects, with a particular focus on oxytocin (OXT), a crucial neuropeptide for social bonding and emotion regulation (Olff et al., 2013).

OXT is synthesized by the paraventricular nucleus (PVN) and supraoptic nucleus (SON) of the hypothalamus and released into the central nervous system (CNS) and endocrine system to exert its effects (Meyer-Lindenberg et al., 2011). In the endocrine system, OXT is secreted by the posterior pituitary into the bloodstream to support reproductive processes like uterine contraction and lactation (Grewen et al., 2010; Maud et al., 2018). In the CNS, OXT is released from dendritic or axonal terminals to specific brain regions to modulate neurotransmitter release, synaptic plasticity, and neural network activity (Chini et al., 2017; Jurek and Neumann, 2018). For example, OXT promotes the release of γ-aminobutyric acid (GABA), which reduces neuronal excitability and produces anxiolytic effects (Sobota et al., 2015; Yuki, 2023). OXT engages in intracellular signaling to regulate gene expression (Jurek and Neumann, 2018; Uvnas-Moberg, 2024) and synaptic plasticity in the amygdala (Gur et al., 2014), hippocampus (Lin and Hsu, 2018), and prefrontal cortex (Ninan, 2011) to support long-term adaptations in emotional responses and social behaviors (Marlin and Froemke, 2017). OXT can suppress the expression of corticotropin-releasing hormone (CRH) and reduce downstream cortisol secretion to moderate stress responses (Bowling, 2023; Harvey, 2020; Jurek and Neumann, 2018).

OXT also plays a role in social cognition (Harvey, 2020). It enhances an individual's ability to recognize, process, and respond to social cues. Notably, these effects are influenced by contextual factors (e.g., safe or threatening environments), and individual factors, including sex, hormonal status, gene variations, attachment style, history of childhood trauma, and the presence of psychiatric disorders, influence the OXTergic system responsiveness (Olff et al., 2013). These findings suggest that the role of OXT is to enhance sensitivity to environmental changes for appropriate behavioral selection (Steinman et al., 2019).

It is unclear how music modulates the OXTergic system to influence emotion regulation and social bonding. Although empirical studies have suggested that music can lead to peripheral OXT changes, the findings are inconsistent. Some studies reported OXT increases after musical activities (Grape et al., 2003; Nilsson, 2009), while others observed no significant change (Palumbo et al., 2022) or even decreases (Eerola et al., 2021; Fancourt et al., 2016; Schladt et al., 2017). The discrepancies may arise from differences in participant characteristics, types of music-based intervention, study designs, OXT measurements, and contextual factors (Engel et al., 2019; MacLean et al., 2019; Tabak et al., 2023). Besides, most studies have been conducted in laboratory or clinical settings with limited ecological validity, which raises questions about whether music-induced OXT changes observed under controlled conditions can be generalized to real-life contexts, especially collective musical rituals.

To address the gaps, this systematic review synthesizes current evidence on how music modulates the OXTergic system in humans. By considering the contextual factors, population characteristics, and intervention features, the review refines the theoretical model of music-induced OXT response to offer guidance to optimize music-based interventions for populations with social impairments. For a rapidly developing field, it will provide a reliable foundation for future research and clinical applications.

The objective of the current review is to explore how individual and group musical activities influence mental health through the OXTergic system in terms of social bonding and emotion regulation. We will address the following questions:

(1) How do OXT levels change in different populations in music-based interventions?
(2) Which characteristics of music-based interventions can be associated with significant changes in OXT levels and psychosocial outcomes?
(3) What are the strengths and limitations of OXT measurements in music-based interventions?

## 2 Methods

### 2.1 Search strategy

The systematic review was conducted in accordance with the Preferred Reporting Items for Systematic Reviews and Meta-Analyses (PRISMA) 2020 guidelines. Literature searches were conducted on November 23, 2024, in five databases: PubMed, Embase, Scopus, Web of Science Core Collection, and PsycInfo. Search terms combined keywords related to music, ritual, OXT, emotion regulation, and social bonding. A detailed search strategy is presented in the Supplementary material S1.

### 2.2 Eligibility criteria

Studies were included if they met the following criteria: (1) original, peer-reviewed, full-text articles published in English up to October 2024; (2) study designs were randomized controlled trials (RCTs), quasi-experimental designs, or observational studies; (3) populations including healthy individuals, clinical populations, or both; (4) interventions involving music therapy or musical activities (e.g., music listening, singing, or playing instruments) delivered individually or in groups; and (5) outcomes reporting OXT measurements along with at least one psychosocial, clinical, or subjective outcome. Exclusion criteria included: (1) non-human studies; (2) reviews or editorials without original data; (3) studies focusing solely on pharmacotherapy (unless compared to music-based interventions); (4) conference abstracts or book chapters; and (5) non-peer-reviewed papers. There were no restrictions regarding participant age, sample size, geographical location, or comparator.

### 2.3 Study selection

All identified records were imported into reference management software, and duplicates were removed. Titles and abstracts were screened independently by the first author. Full texts of potentially relevant articles were then assessed for eligibility. Uncertainties were resolved through discussion with the second author.

### 2.4 Quality assessment

The quality of included studies was assessed using the Joanna Briggs Institute (JBI) critical appraisal tools for RCTs and for quasi-experimental studies. The tools evaluate the validity of study design, methodology, statistical conclusions, and risk of bias (Barker et al., 2024, 2023). The quality assessment was conducted by the first author and verified by the second author.

### 2.5 Data extraction, analysis, and synthesis

From the full texts of selected articles and their supplementary material, one reviewer extracted the data, including study characteristics, participant characteristics, intervention details, outcome measures, results, and statistical analyses.

Quantitative OXT changes were transformed into qualitative categories: increase, decrease, or no significant change. Psychosocial outcomes were grouped into broader domains: anxiety, depression, empathy, social bonding, trust, stress and relaxation, as well as emotion and mood. Contextual descriptions were extracted and categorized into four dimensions: (1) nature of the activity (e.g., passive listening, active music-making, or music therapy); (2) stress signals (e.g., levels of physical or mental stress); (3) social cues (e.g., presence of others); and (4) familiarity (e.g., familiarity with the music, participants, or environments).

The reviewer conducted a framework synthesis and a narrative synthesis. The framework synthesis was based on the model proposed by Bowling (2023), following a five-stage process (Brunton et al., 2020): familiarization, framework selection, indexing, charting, and mapping/interpretation. The narrative synthesis was used to summarize patterns within and across studies, explore relationships, and assess the robustness of findings in terms of methodological quality (Impellizzeri and Bizzini, 2012). Finally, a convergent design (Hong et al., 2017) was used to integrate quantitative and qualitative data.

## 3 Results

### 3.1 Study selection and study characteristics

**Figure 1:**
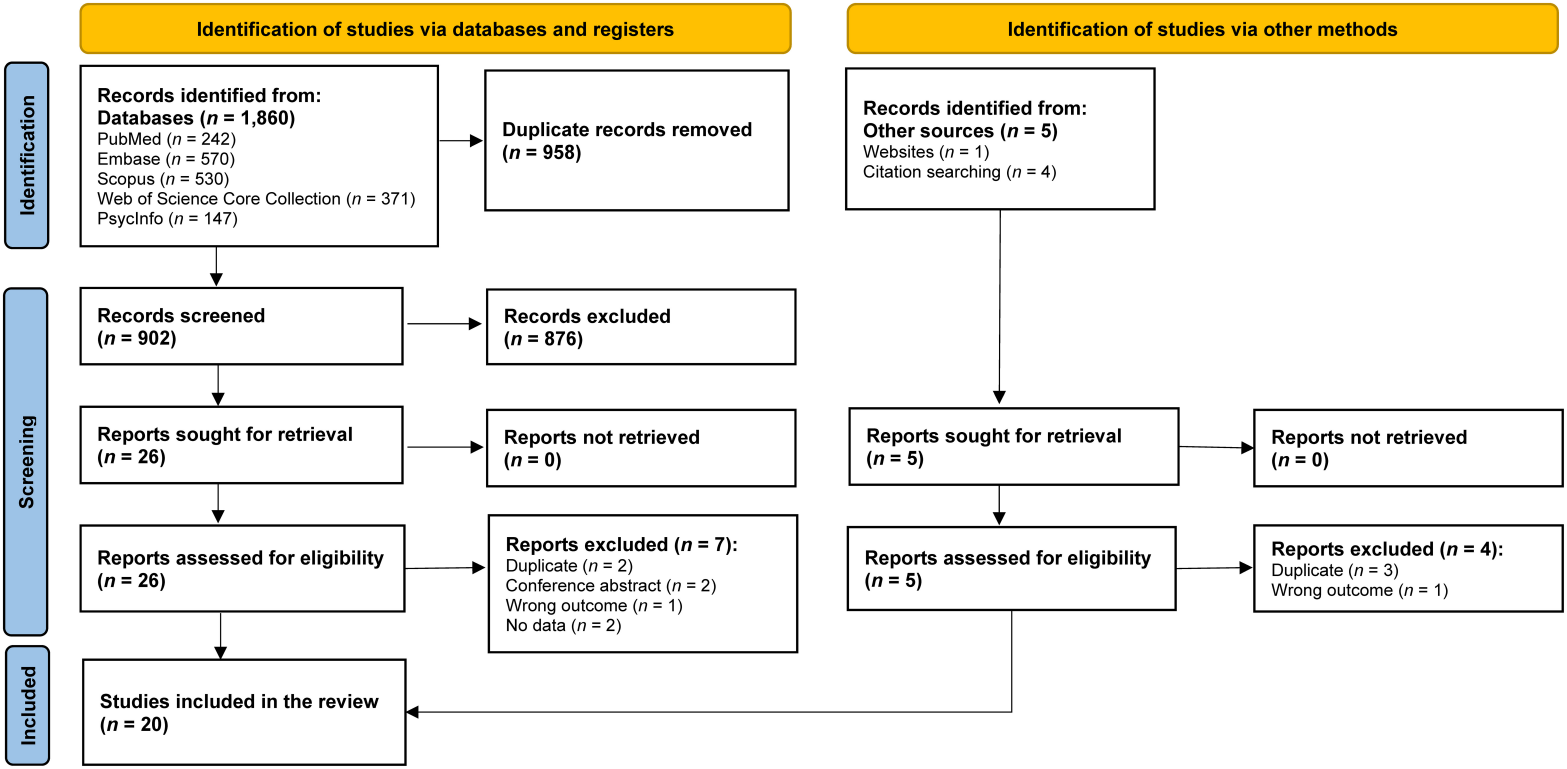
PRISMA flow diagram.

A PRISMA flow diagram is presented in Figure 1, showing study identification and inclusion of eligible studies. A total of 1,865 records were identified, of which 1,860 were from electronic databases, and five were from other sources. After removing duplicates, 902 records were screened based on titles and abstracts. Thirty-one full-text articles were assessed for eligibility, and 11 studies were excluded (five duplicates, two conference abstracts, two with wrong outcomes, and two without OXT data). Finally, 20 studies (seven RCTs and 13 quasi-experiments) were included in the systematic review.

Table 1 summarizes the characteristics of the included studies, which were published between 2003 and 2023 across 11 countries. The majority of the studies were conducted in Europe (*n* = 12, 60%), followed by North America (*n* = 5, 25%), and Asia (*n* = 3, 15%). All studies comprised 877 participants. Sample sizes ranged from 4 to 193 participants (median = 25.5).

**Table 1:** Study characteristics.

**Author (year)**	**Study design**	**Country**	**Sample**	**Intervention and comparison**	**Music genre**	**Outcome**	**Finding**
Bowling et al. (2022)	Quasi-experiment	Austria	71 healthy adults (26 M, 45 F) aged 17–28 (mean: 23.1 years); Some participants took part in more than one intervention	One session, 20 min: Singing together (*n* = 37); Singing alone (*n* = 25); Speaking together (*n* = 31); Speaking alone (*n* = 19)	Classical choral (“The Armed Man” by Karl Jenkins)	Salivary OXT levels; PANAS; IOS scale	OXT decreased less in both group and solo singing compared to group and solo speaking, indicating a stronger role of singing in promoting social bonding.
Corbett et al. (2011)	Quasi-experiment	USA	8 ASD children (7 M, 1 F) aged 6–17 (mean: 11.3 years)	38 rehearsals from 1 day/week increasing to 3–4 days/week, 2 h per session, for 3 months: SENSE therapy	Children's song (Musical theatrical production of Disney's “The Jungle Book”)	Plasma OXT levels; NEPSY-memory for faces; NEPSY-affect recognition; NEPSY-theory of mind	SENSE participants showed improvement in face identification and theory of mind skills without significant changes in baseline OXT levels after a long-term intervention.
Dai et al. (2012)	Quasi-experiment	USA	21 adults: 13 WS (6 M, 7 F) aged 19–42 (mean: 30.2 years); 8 TC (4 M, 4 F) aged 19–45 (mean: 29.4 years)	One session, 2 h, with stimuli at specific time points: The favorite music that elicited positive emotions, lasted 5–8 min; Cold water at 15 °C, lasted less than 45 s	Chosen by the participants	Serum OXT levels; Serum AVP levels; Adolph's approachability; Salk Institute Sociability Questionnaire; Scales of Independent Behavior-Revised	Compared with TC group, music and cold stimuli cause an exaggerated release of OXT and AVP in WS group. Higher basal OXT levels were correlated with increased approach to strangers and decreased adaptive social behaviors in WS patients.
Eerola et al. (2021)	Crossover quasi-experiment	Finland	62 healthy women aged 18–39, divided into high-empathy (*n* = 32; mean: 24.2 years) and low-empathy (*n* = 30; mean: 24.9 years) groups	One session, 60 min: Listening to unfamiliar sad instrumental music, 20 min; Silence, 20 min	Classical (Mozart's Piano Concerto No. 23 2nd movements, Kamen's “Discovery of the Camp,” and Piazzola's “Oblivion”)	Plasma OXT levels; Profile of mood states; VAS for felt pleasure, being moved, anxiousness, relaxation, and sadness	High-empathy participants experienced greater positive mood and feelings of being moved during music exposure compared with the low-empathy participants. In the high-empathy group, OXT levels were significantly lower in the music condition than in the silence condition.
Fancourt et al. (2016)	Quasi-experiment	UK	193 adults (20% M, 80% F): 55 cancer patients (mean age: 60.8 years); 72 current carers (mean age: 56.9 years); 66 bereaved carers (mean age: 59.7 years)	One session, 70 min: Choir rehearsal with live music	Unclear	Salivary OXT levels; Salivary beta-endorphin levels; Salivary cortisol levels; VAS of mood scales; VAS of stress scales; VAS of connectedness	Choir singing reduced salivary OXT, beta-endorphin, and cortisol levels, and increased cytokine activity. Participants with worse mental health experienced greater emotional improvement after singing.
Filippa et al. (2021)	Crossover RCT	Italy	20 preterm infants (11 M, 9 F) in the NICU: stable medical condition; mean gestational age at test: 34.8 weeks; mean weight at test: 2264 g	Preterm infants received 3 conditions during painful procedures, each for 10 min: Maternal singing; Maternal speaking; Standard care	Unclear	Salivary OXT levels; Premature Infant Pain Profile	For preterm infants, maternal speech significantly reduced pain scores and increased OXT levels. Maternal singing led to marginally significant increases in OXT levels but did not significantly reduce pain scores.
Filippa et al. (2023)	Crossover RCT	Italy	20 mothers of preterm infants, aged 23–34 (mean: 29.2 years)	2 sessions, each for 10 min: Maternal singing to the baby; Maternal speaking to the baby	Unclear	Salivary OXT levels; STAI-S	Maternal singing and speaking during their infant's painful procedures significantly increased maternal salivary OXT levels and reduced maternal anxiety.
Good and Russo (2022)	Crossover quasi-experiment	Canada	8 healthy older adults (1 M, 7 F): cognitively intact; mean age: 72.8 years	2 sessions, each for 45 min: Group singing led by a director; Individual singing led by pre-recorded choir activities	Unclear	Salivary OXT levels; PANAS	Group singing triggered the OXT release and improved moods, which may result from social factors rather than from individual factors.
Grape et al. (2003)	Quasi-experiment	Sweden	16 adults: 8 professional singers (4 M, 4 F) aged 26–49 (mean: 36.4 years); 8 amateur singers (2 M, 6 F) aged 28–53 (mean: 40.2 years)	One session, 45 min: A classical singing lesson with the participants' teachers	Classical	Serum OXT levels; VAS for the dimensions: sad-joyful, anxious-calm, worried-elated, listless-energetic, tense-relaxed	Both amateurs and professionals showed OXT increases after singing. A singing lesson promoted wellbeing in amateurs but not in professionals.
Jezova et al. (2013)	Crossover quasi-experiment	Slovak	14 healthy males aged 21–29	2 sessions (washout: 7 days), each for 45 min: Listening to pleasant rock music (played forward) during stress tasks; Listening to unpleasant rock music (played backward) during stress tasks	Rock (Music excerpts by Rolling Stones)	Plasma OXT levels; Plasma ACTH levels; Plasma cortisol levels; Plasma AVP levels; STAI-S	The acute increase in state anxiety induced by unpleasant music modulated physiological responses under stress conditions, with reduced HPA activity and increased OXT and AVP levels, while cortisol levels remained unchanged.
Keeler et al. (2015)	Quasi-experiment	USA	4 university students (2 M, 2 F) aged ≥18; Jazz vocalists	One session, including 2 performances (washout: 30 min): Standard group singing, 5.6 min; Improvised group singing, 6 min	Jazz (A pre-composed song by the researcher)	Plasma OXT levels; Plasma ACTH levels; Flow State Scale-2	Both standard and improvised group singing induced social flow in participants. OXT levels decreased in the standard performance, but increased in the improvised condition.
Kreutz (2014)	Quasi-experiment	Germany	21 adults (5 M,16 F) aged 18–65 (Median: over 50 years); Mixed group of novice and experienced chorists, with 9 participants reporting chronic diseases	10 sessions, 30 min per session: Choral singing (7th session); Chatting (8th session)	Folk rock (four-part choral arrangements of “California Dreamin”)	Salivary OXT levels; *Ad hoc* questionnaire on positive and negative feelings	Singing and chatting significantly increased positive feelings. Salivary OXT levels increased significantly and negative feelings were reduced more after choral singing but not after chatting.
Maramis et al. (2021)	Cluster RCT	Indonesia	61 healthy fourth-grade elementary students: Playing angklung (12 M, 8 F); Practicing silence (9 M, 12 F); Control group (11 M, 9 F)	15 min every day, for 8 weeks, before morning classes: Playing angklung; Practicing silence; Control: no intervention	Indonesian traditional music (national and regional songs)	Salivary OXT levels; PANAS-Child Form	Salivary OXT levels significantly increased in the silence group compared to those in the control group, without significant changes in clinical parameters. A slight decrease in OXT level was observed in the angklung group, possibly due to the cognitive demands of decoding, memorizing, and playing musical notes. The control group showed the greatest OXT decrease.
Nilsson (2009)	RCT	Sweden	40 patients who have undergone heart surgery on postoperative day one: Music group (17 M, 3 F; mean age: 64 years); Control group (15 M, 5 F; mean age: 67 years)	One session, 60 min: Music group: listening to 30-min soft, relaxing music during bed rest and then 30-min standard bed rest; Control group: 60-min standard bed rest	Ambient (MusiCure)	Serum OXT levels; Relaxation numeric rating scale	Baseline serum OXT levels and relaxation scores were significantly lower in the music group than in the control group, likely due to differences in surgical duration and type, but no differences were observed after intervention. Music during bed rest increased OXT levels, whereas bed rest alone decreased them, suggesting music-related psychological processes may promote OXT release.
Ooishi et al. (2017)	Quasi-experiment	Japan	26 healthy males aged 21–34 (mean: 29.4 years); Without musical training and a habit of listening to classical music	2 sessions, each for 20 min: Listening to slow-tempo music sequences; Listening to fast-tempo music sequences	Classical (Piano pieces by Chopin)	Salivary OXT levels; Salivary cortisol levels; Heart rate variability	Listening to slow-tempo music significantly increased salivary OXT levels and lnHF, and significantly decreased the heart rate, suggesting an involvement of vagal activity. No correlation was found between OXT changes and self-reported relaxation.
Palumbo et al. (2022)	RCT	USA	25 adults with post-stroke unilateral hemiparesis: Predominantly Black; MULT-I (5 M, 8 F; mean age: 61.2 years); HEP (8 M, 4 F; mean age: 61.8 years)	45 min per session, twice a week, for 6 weeks: Music Upper Limb Therapy-Integrated (MULT-I); Home exercise program (HEP)	Unclear	Serum OXT levels; Serum BDNF levels; Patient Health Questionnaire-9	MULT-I significantly reduced depression levels and increased BDNF levels compared with HEP. There were no significant changes in serum OXT levels in both groups.
Riedl et al. (2017)	RCT	Austria	30 healthy male graduate and doctoral students: Music group (*n* = 15; mean age: 27.2 years); Control group: (*n* = 15; mean age: 26.1 years)	One session including 40 rounds of the trust game: Music group: listening to music during the game; Control group: no music	Classical (Schubert's “Marche Militaire”)	Plasma OXT levels; Investment amount in the trust game; Trustworthiness 7-point Likert scale	Music did not have significant effects on OXT levels, trust behavior, or perceived trustworthiness. In the no-music condition, OXT increase was associated with greater perceived trustworthiness but did not influence trust behavior.
Schladt et al. (2017)	Quasi-experiment	Germany	38 healthy adults: Cohort 1 (9 M, 12 F) aged 19–26 (median: 22 years); Cohort 2 (8 M, 9 F) aged 18–29 (median: 23 years); Student chorists	2 sessions, each for 20 min: Cohort 1: A-B (washout: 2 days) or B-A (washout: 4 days); Cohort 2: A-B (washout: 4 days); A: choral singing, B: solo singing	Classical (Cohort 1: Handel's “Messiah”; Cohort 2: Bach's “Christmas Oratorio”)	Salivary OXT levels; STADI-S	Both choral and solo singing improved moods. Choral singing reduced OXT levels, while solo singing modestly increased OXT levels, which may be caused by increased or decreased stress signals.
Wulff et al. (2021)	RCT	Germany	172 pregnant women aged 18–42 (mean: 34.0 years): Music group (*n* = 64; gestational age: 31.8 weeks); Singing group (*n* = 59; gestational age: 30.6 weeks); Control group (*n* = 49; gestational age: 32.7 weeks)	Music: one 30-min session of music listening with a therapist, and home-based music listening for 10–15 min daily until childbirth; Singing: 30-min singing sessions with a therapist 2–4 times, and home-based singing for 10–15 min daily until childbirth; Control: no intervention	Listening group: Classical; Singing group: Children's song	Salivary OXT levels; Self-Assessment Manikin; VAS of perceived closeness; Maternal Antenatal Attachment Scale; The Edinburgh Postnatal Depression Scale	There were significant increases in salivary OXT levels and reductions in salivary cortisol levels in the singing and music groups. Compared to the music group, the singing group showed greater improvements in emotions, cortisol levels, and perceived closeness to the unborn child. No significant effects were found on depressive symptoms or bonding questionnaire scores.
Yuhi et al. (2017)	Quasi-experiment	Japan	27 maltreated children (22 M, 5 F) aged 8–15	Group taiko drumming, less than once a week, for 18 months: Recital: 12 times, 5–60 min (mean: 14.1 min); Practice: 5 times, 80–155 min (mean: 108 min); Free condition: 6 times, 90–200 min (mean: 118 min)	Japanese kumi-daiko	Salivary OXT levels; Personalities recorded by teachers	Group drumming supported emotional and social improvements in maltreated children, but significant OXT increases were observed only in elementary boys in recital sessions.

ACTH, adrenocorticotropic hormone; ASD, autism spectrum disorder; AVP, arginine vasopressin; BDNF, brain-derived neurotrophic factor; F, female; HPA axis, hypothalamic-pituitary-adrenal axis; IOS, Inclusion of Other in the Self; lnHF, logarithmic transformation of low frequency powers of heart rate variability; M, male; NEPSY, A Developmental Neuropsychological Assessment; OXT, oxytocin; PANAS, Positive and Negative Affect Schedule; RCT, randomized controlled trial; SENSE, Social Emotional NeuroScience Endocrinology; STADI-S, the state subscale of State-Trait Anxiety-Depression Inventory; STAI-S, the state subscale of State-Trait Anxiety Inventory; TC, typical control; VAS, visual analog scale; WS, Williams syndrome.

The reviewed music-based interventions encompassed singing, playing instruments, listening to music, and music therapy. Most studies used pieces of classical music as materials. Only two studies adopted quasi-ritualistic music-based interventions grounded in culture: one study from Japan used group taiko drumming (Yuhi et al., 2017), and one study from Indonesia examined a school-based intervention program combining Indonesian traditional music (Maramis et al., 2021).

One reviewer used the JBI critical appraisal tools to assess study quality. Among the studies, 17 were rated as low risk of bias, two as moderate risk, and one as high risk. Tables 2, 3 present the detailed results of the quality assessment. No studies were excluded based on quality alone.

**Table 2:** Quality assessment for RCTs.

**Author (year)**	**1. Was true randomization used for the assignment of participants to treatment groups?**	**2. Was allocation to treatment groupsconcealed?**	**3. Were treatment groups similar at thebaseline?**	**4. Were participants blind to treatment assignment?**	**5. Were those delivering the treatment blind to treatment assignment?**	**6. Were treatment groups treated identically other than the intervention of interest?**	**7. Were outcome assessors blind to treatment assignment?**	**8. Were outcomes measured in the same way for treatment groups?**	**9. Were outcomes measured in a reliable way?**	**10. Was follow-up complete and if not, were differences between groups in terms of their follow-up adequately described and analyzed?**	**11. Were participants analyzed in the groups to which they were randomized?**	**12. Was appropriate statistical analysisused?**	**13. Was the trial design appropriate and any deviations from the standard RCT design accounted for in the conduct and analysis of the trial?**
Filippa et al. (2021) ^a^	Yes	Yes	Yes	N/A	N/A	Yes	No	Yes	Yes	Yes	Yes	Yes	Yes
Filippa et al. (2023) ^a^	Yes	Yes	Yes	N/A	N/A	Yes	No	Yes	Yes	Yes	Yes	Yes	Yes
Maramis et al. (2021)	Yes	Yes	Yes	N/A	N/A	Yes	Unclear	Yes	Yes	Yes	Yes	Yes	Yes
Nilsson (2009)	Yes	Yes	No	N/A	N/A	Yes	No	Yes	Yes	Yes	Yes	Yes	Yes
Palumbo et al. (2022)	Yes	Yes	No	N/A	N/A	Yes	Unclear	Yes	Yes	Yes	Yes	Yes	Yes
Riedl et al. (2017)	Yes	Yes	Yes	N/A	N/A	Yes	No	Yes	Yes	Yes	Yes	Yes	Yes
Wulff et al. (2021)	Yes	Yes	No	N/A	N/A	Yes	No	Yes	Yes	Yes	Yes	Yes	Yes

Question 4 and question 5 were marked as N/A because blinding participants and treatment providers was not feasible. For RCTs, studies scoring ≥ 9 on the 13-question checklist were considered high quality, while those scoring < 4 were deemed low quality.

^a^The two articles were the same clinical trial: Filippa et al. (2021) reported data from preterm infants, and Filippa et al. (2023) focused on the mothers of the preterm infants.

**Table 3:** Quality assessment for quasi-experimental designs.

**Author (year)**	**1. Is it clear in the study what is the cause and what is the effect?**	**2. Was there a control group?**	**3. Were participants included in any comparisons similar?**	**4. Were the participants included in any comparisons receiving similar treatment/care, other than the exposure or intervention of interest?**	**5. Were there multiple measurements of the outcome, both pre- and post-intervention/ exposure?**	**6. Were the outcomes of participants included in any comparisons measured in the same way?**	**7. Were outcomes measured in a reliable way?**	**8. Was follow-up complete and if not, were differences between groups in terms of their follow-up adequately described and analyzed?**	**9. Was appropriate statistical analysis used?**
Bowling et al. (2022)	Yes	No	Yes	Yes	Yes	Yes	Yes	Yes	Yes
Corbett et al. (2011)	Yes	No	Yes	Unclear	Yes	Yes	Yes	Yes	Yes
Dai et al. (2012)	Yes	No	No	Yes	Yes	Yes	Yes	Yes	Yes
Eerola et al. (2021)	Yes	Yes	No	Yes	Yes	Yes	Yes	Yes	Yes
Fancourt et al. (2016)	Yes	No	No	Yes	Yes	Yes	Yes	Yes	Yes
Good and Russo (2022)	Yes	No	Yes	Yes	Yes	Yes	Yes	No	Yes
Grape et al. (2003)	Yes	No	No	No	Yes	Yes	Unclear	Yes	Yes
Jezova et al. (2013)	Yes	No	Yes	Yes	Yes	Yes	Yes	Yes	Yes
Keeler et al. (2015)	Yes	No	Yes	Yes	Yes	Yes	Unclear	Yes	Yes
Kreutz (2014)	Yes	No	Yes	Yes	Yes	Yes	Unclear	No	No
Ooishi et al. (2017)	Yes	No	Yes	Yes	Yes	Yes	Yes	Yes	Yes
Schladt et al. (2017)	Yes	No	Yes	Yes	Yes	Yes	Yes	Yes	Yes
Yuhi et al. (2017)	Yes	No	No	No	Yes	Yes	No	No	No

For quasi-experimental studies, those scoring ≥ 7 on the 9-question checklist were rated as high quality, and those scoring < 4 were considered low quality.

### 3.2 Population characteristics and OXT responses

The included studies encompassed diverse populations, with a primary focus on healthy adults. Other studies examined clinical populations, including cancer patients and their carers Fancourt et al., (2016), pregnant women Wulff et al., (2021), preterm infants Filippa et al., (2021) and their mothers Filippa et al., (2023), individuals with Williams syndrome Dai et al., (2012), post-surgical patients (Nilsson, 2009), post-stroke patients (Palumbo et al., 2022), children with autism spectrum disorder (ASD; Corbett et al., 2011), and maltreated children (Yuhi et al., 2017). Two studies (Grape et al., 2003; Kreutz, 2014) recruited mixed samples that varied in age, sex, health status, and singing experiences, as detailed in Table 1.

Participant ages ranged from 34.8 gestational weeks (preterm infants) to 72.8 years (healthy older adults). Most studies had a predominance of female participants. Baseline peripheral OXT levels showed notable differences between populations. In typical control individuals, the patterns of plasma OXT response to music demonstrated low inter-individual variability (Dai et al., 2012), although they had different baseline OXT levels. By contrast, patients with Williams syndrome exhibited higher baseline plasma OXT levels and greater variability in response to music Dai et al., (2012). In pregnant and postpartum mothers (Filippa et al., 2023; Wulff et al., 2021), baseline peripheral OXT levels were low, with small yet statistically significant changes after musical activities. The patterns of OXT response to music were not influenced by sex (Bowling et al., 2022; Filippa et al., 2021). Other covariates the phase of the menstrual cycle, the use of hormonal contraception, and the musical sophistication index did not change the patterns (Eerola et al., 2021).

Tables 4, 5 summarize the peripheral OXT changes induced by music across populations and contexts (also see Supplementary material S2). These results revealed the context-dependence of OXT responses to music. After group singing, OXT levels decreased in healthy young adults Bowling et al., (2022); Keeler et al., (2015); Schladt et al., (2017) but increased in healthy older adults Good and Russo, (2022). OXT reductions were observed in cancer patients and their carers after choral singing Fancourt et al., (2016). For preterm infants Filippa et al., (2021) and their mothers Filippa et al., (2023), as well as pregnant women Wulff et al., (2021), maternal singing to infants or fetuses increased their OXT levels. Listening to slow-tempo music generally triggered OXT release in several populations, including healthy males Ooishi et al., (2017), pregnant women with trait anxiety Wulff et al., (2021), and post-surgical patients Nilsson, (2009). Interestingly, listening to sad music led to significant OXT decreases in healthy females with high empathy but not in healthy females with low empathy Eerola et al., (2021). However, in children with ASD Corbett et al., (2011) and post-stroke patients Palumbo et al., (2022), no significant changes in peripheral OXT levels were observed after long-term group music therapy.

**Table 4:** Peripheral OXT changes induced by music in healthy populations.

**Population**	**Peripheral OXT response in different contexts**						
	**Group singing**	**Improvised group singing**	**Solo singing**	**Listening to slow music**	**Listening to fast music**	**Listening to sad music**	**Playing the instruments in a group**
**Healthy young adults**							
Bowling et al. (2022)	↓		↓				
Keeler et al. (2015)	↓	↑					
Schladt et al. (2017)	↓		→				
**Healthy females with high empathy**							
Eerola et al. (2021)						↓	
**Healthy females with low empathy**							
Eerola et al. (2021)						→	
**Healthy males**							
Ooishi et al. (2017)				↑	→		
Riedl et al. (2017)					→		
Jezova et al. (2013)					↓ or ↑^a^		
**Healthy older adults**							
Good and Russo (2022)	↑		→				
**Healthy children**							
Maramis et al. (2021)							→^b^

↑ = significant increase; ↓ = significant decrease; → = no significant change. Significance levels (e.g., *p* < 0.05, *p* < 0.01, *p* < 0.001) are not distinguished in the review. All significance is defined as *p* < 0.05.

^a^Participants showed OXT decreases after listening to pleasant rock music during stress tasks, but showed OXT increases after listening to unpleasant rock music during stress tasks.

^b^OXT measurements were obtained before and after a long-term program.

**Table 5:** Peripheral OXT changes induced by music in clinical populations.

**Population**	**Peripheral OXT responses in different contexts**						
	**Group singing**	**Classical singing lesson**	**Maternal singing to a baby**	**Listening to slow music**	**Listening to the favorite positive music**	**Playing the instruments in a group**	**Group music therapy**
**Williams Syndrome (WS)**							
Dai et al. (2012)					↑		
**Typical controls paired with WS**							
Dai et al. (2012)					↑		
**Cancer patients**							
Fancourt et al. (2016)	↓						
**Carers of cancer patients**							
Fancourt et al. (2016)	↓						
**Pregnancy with trait anxiety**							
Wulff et al. (2021)			↑	↑			↑
**Mothers of preterm infants**							
Filippa et al. (2023)			↑				
**Preterm infants**							
Filippa et al. (2021)			↑^c^				
**Post-surgical patients**							
Nilsson (2009)				↑			
**Children with autism spectrum disorder**							
Corbett et al. (2011)							→^e^
**Maltreated boys**							
Yuhi et al. (2017)						↑^d^	
**Post-stroke patients**							
Palumbo et al. (2022)						→^e^	→^e^
**Mixed sample**							
Grape et al. (2003)		↑^b^					
Kreutz (2014)	↑^a^						

↑ = significant increase; ↓ = significant decrease; → = no significant change. Significance levels (e.g., *p* < 0.05, *p* < 0.01, *p* < 0.001) are not distinguished in the review. All significance is defined as *p* < 0.05.

^a^The sample included mixed age, sex, clinical conditions, and singing experiences.

^b^The sample included mixed age, sex, and singing experiences.

^c^The change was marginally significant.

^d^There was a high risk of bias due to inconsistency in the duration of intervention and control conditions, the measurement time points, the sample size, and the number of sessions.

^e^The measurements were obtained before and after a long-term program.

### 3.3 Psychosocial outcomes and OXT changes

Across the included studies, associations between psychosocial outcomes and peripheral OXT changes varied by population, context, and measurement time point. Table 6 summarizes the relationships between psychosocial outcomes, OXT changes, and context characteristics.

**Table 6:** Relationships between psychosocial outcomes, OXT changes, and context characteristics.

**Author (year)**	**Population**	**Psychosocial outcome**		**OXT response**		**Context**			
		**Measure**	**Change**	**Measure**	**Change**	**Activity**	**Stress signal**	**Social cue**	**Familiarity**
**Anxiety**									
Schladt et al. (2017)	Healthy young adults	STADI-S	↓	Salivary OXT levels	↓	Choral singing	Possible	Limited	High
Schladt et al. (2017)	Healthy young adults	STADI-S	↓	Salivary OXT levels	→	Solo singing	Possible	No	High
Jezova et al. (2013)	Healthy males	STAI-S	↑	Plasma OXT levels (iAUC)	↑	Listening to unpleasant rock music during stress tasks	High	No	Low
Jezova et al. (2013)	Healthy males	STAI-S	→	Plasma OXT levels (iAUC)	↓	Listening to pleasant rock music during stress tasks	High	No	Moderate
Filippa et al. (2023)	Mothers of preterm infants	STAI-S	↓	Salivary OXT levels	↑	Maternal singing	Possible	Yes	Low
**Depression**									
Schladt et al. (2017)	Healthy young adults	STADI-S	↓	Salivary OXT levels	↓	Choral singing	Possible	Limited	High
Schladt et al. (2017)	Healthy young adults	STADI-S	↓	Salivary OXT levels	→	Solo singing	Possible	No	High
Palumbo et al. (2022)	Post-stroke patients	PHQ-9	↓	Plasma OXT levels	→	Group music therapy (MULT-I), 6 weeks	Moderate	Yes	Increased gradually
**Empathy**									
Eerola et al. (2021)	Healthy females with high empathy	VAS-being moved	67.2/100^a^	Plasma OXT levels	↓	Listening to unfamiliar sad instrumental music	Possible	No	Low
Eerola et al. (2021)	Healthy females with low empathy	VAS-being moved	52.3/100^a^	Plasma OXT levels	→	Listening to unfamiliar sad instrumental music	Possible	No	Low
Corbett et al. (2011)	ASD children	NEPSY-affect recognition	→	Plasma OXT levels	→	Musical theater therapy, 3 months	Moderate	Yes	Increased gradually
Corbett et al. (2011)	ASD children	NEPSY-theory of mind	↑	Plasma OXT levels	→	Musical theater therapy, 3 months	Moderate	Yes	Increased gradually
**Social bonding**									
Bowling et al. (2022)	Healthy young adults	IOS scale	↑	Salivary OXT levels	↓	Choral singing	Possible	Yes	High
Keeler et al. (2015)	Jazz vocalists	FSS-2	Presence of social flow^a^	Plasma OXT levels	↓	Standard choral singing	Moderate	Yes	Low
Keeler et al. (2015)	Jazz vocalists	FSS-2	Presence of social flow^a^	Plasma OXT levels	↑	Improvised choral singing	High	Yes	Low
Fancourt et al. (2016)	Cancer patients	VAS-connectedness	↑	Salivary OXT levels	↓	Choral singing	Possible	Yes	Moderate
Fancourt et al. (2016)	Carers of cancer patients	VAS-connectedness	↑	Salivary OXT levels	↓	Choral singing	Possible	Yes	Moderate
Corbett et al. (2011)	ASD children	NEPSY-memory for faces	↑	Plasma OXT levels	→	Musical theater therapy, 3 months	Moderate	Yes	Increased gradually
**Trust**									
Riedl et al. (2017)	Healthy males	Investment amount in the trust game	Higher value^b^	Plasma OXT levels	→	Listening to fast classical music	Possible	Yes	Low
Riedl et al. (2017)	Healthy males	7-point Likert scale of trustworthiness	Higher value^b^	Plasma OXT levels	→	Listening to fast classical music	Possible	Yes	Low
**Stress and relaxation**									
Eerola et al. (2021)	Healthy females with high empathy	VAS- relaxation	79.4/100^a^	Plasma OXT levels	↓	Listening to unfamiliar sad instrumental music	Possible	No	Low
Eerola et al. (2021)	Healthy females with low empathy	VAS- relaxation	62.6/100^a^	Plasma OXT levels	→	Listening to unfamiliar sad instrumental music	Possible	No	Low
Nilsson (2009)	Post-surgical patients	Relaxation numeric rating scale	↑	Serum OXT levels	↑	Listening to soft, relaxing, slow music	Low	No	Low
Grape et al. (2003)	Mixed sample-amateur singers	VAS-tense-relaxed	Relaxation ↑	Serum OXT levels	↑	Classical singing lesson	Moderate	Yes	High
Grape et al. (2003)	Mixed sample-professional singers	VAS-tense-relaxed	Relaxation ↑	Serum OXT levels	↑	Classical singing lesson	Moderate	Yes	High
Fancourt et al. (2016)	Cancer patients	VAS-stress	↓	Salivary OXT levels	↓	Choral singing	Possible	Yes	Moderate
Fancourt et al. (2016)	Carers of cancer patients	VAS-stress	↓	Salivary OXT levels	↓	Choral singing	Possible	Yes	Moderate
**Emotion and mood**									
Bowling et al. (2022)	Healthy young adults	PANAS (positive affect)	↑	Salivary OXT levels	↓	Choral singing	Possible	Yes	High
Bowling et al. (2022)	Healthy young adults	PANAS (positive affect)	↑	Salivary OXT levels	↓	Solo singing	Possible	No	High
Good and Russo (2022)	Healthy older adults	PANAS (mood rating)	↑	Salivary OXT levels	↑	Choral singing	Unclear	Yes	High
Good and Russo (2022)	Healthy older adults	PANAS (mood rating)	→	Salivary OXT levels	→	Solo singing	Unclear	No	Moderate
Maramis et al. (2021)	Healthy children	PANAS-C	→	Salivary OXT levels	→	Playing the angklung, 8 weeks	Moderate	Yes	Increased gradually
Eerola et al. (2021)	Healthy females with high empathy	POMS (positive mood)	↑	Plasma OXT levels	↓	Listening to unfamiliar sad music	Possible	No	Low
Eerola et al. (2021)	Healthy females with low empathy	POMS (positive mood)	→	Plasma OXT levels	→	Listening to unfamiliar sad music	Possible	No	Low
Fancourt et al. (2016)	Cancer patients	VAS-mood	↑	Salivary OXT levels	↓	Choral singing	Possible	Yes	Moderate
Fancourt et al. (2016)	Carers of cancer patients	VAS-mood	↑	Salivary OXT levels	↓	Choral singing	Possible	Yes	Moderate
Wulff et al. (2021)	Pregnant mothers with trait anxiety	SAM	Valence ↑, Arousal ↓, Dominance ↑	Salivary OXT levels	↑	Listening to calm classical instrumental music	Low	Yes	Unclear
Wulff et al. (2021)	Pregnant mothers with trait anxiety	SAM	Valence ↑, Arousal ↓, Dominance ↑	Salivary OXT levels	↑	Singing children's songs and lullabies	Low	Yes	Moderate
Grape et al. (2003)	Mixed sample-amateur singers	VAS-anxiousness-calm	Calm ↑	Serum OXT levels	↑	Classical singing lesson	Moderate	Yes	High
Grape et al. (2003)	Mixed sample-professional singers	VAS-anxiousness-calm	Calm →	Serum OXT levels	↑	Classical singing lesson	Moderate	Yes	High
Kreutz (2014)	Participants varied in age, sex, clinical conditions, and singing experiences	*Ad hoc* questionnaire of subjective feelings	Positive feelings ↑, Negative feelings ↓	Salivary OXT levels	↑	Choral singing	Moderate	Yes	Varied in participants

↑ = significant increase; ↓ = significant decrease; → = no significant change. Significance levels (e.g., *p* < 0.05, *p* < 0.01, *p* < 0.001) are not distinguished in the review. All significance is defined as *p* < 0.05.

ASD, autism spectrum disorder; FSS-2, Flow State Scale-2; iAUC, incremental Area Under the Curve; IOS, Inclusion of Other in the Self; MULT-I, Music Upper Limb Therapy-Integrated; NEPSY, A Developmental Neuropsychological Assessment; OXT, oxytocin; PANAS, Positive and Negative Affect Schedule; PANAS-C, Positive and Negative Affect Schedule-Child Form; PHQ-9, Patient Health Questionnaire-9; POMS, Profile of Mood States questionnaire; SAM, Self-Assessment Manikin; STADI-S, the state subscale of State-Trait Anxiety-Depression Inventory; STAI-S, the state subscale of State-Trait Anxiety Inventory; VAS, visual analog scale.

^a^The outcome was measured only once after the intervention.

^b^The outcome showed a higher value in the music condition than in the no-music condition, but the difference was not statistically significant.

Long-term intervention programs did not lead to significant changes in peripheral OXT levels, despite the improvements in psychosocial outcomes. For instance, after participating in interactive improvised group music-making therapy for 6 weeks, post-stroke patients showed improvements in depression and motor functions without significant OXT changes Palumbo et al., (2022). Similarly, a 3-month structured musical theater therapy improved the ASD children's skills of theory of mind and memory for faces, but no significant plasma OXT changes were observed Corbett et al., (2011).

Short-term music-based interventions, such as a single session of singing, often resulted in improvements in anxiety, though not always accompanied by changes in peripheral OXT levels. Maternal singing led to anxiety reduction and OXT increases in mothers of preterm infants Filippa et al., (2023), whereas in other contexts (e.g., healthy adults engaging in choir singing), anxiety reductions occurred accompanied by OXT decreases Schladt et al., (2017). Additionally, unpleasant music stimuli under stress conditions both increased anxiety and plasma OXT levels in healthy males Jezova et al., (2013).

Empathy-related outcomes unveiled noteworthy patterns. For high-empathy healthy females, listening to unfamiliar sad instrumental music elicited stronger self-reported emotional responses, accompanied by significant decreases in plasma OXT levels. In contrast, low-empathy healthy females reported smaller emotional improvements but showed modest OXT increases Eerola et al., (2021).

In an RCT, Riedl et al. (2017) examined whether music listening in a trust game influenced the trust behavior and perceived trustworthiness. During the trust game, no significant changes in OXT levels were observed in the participants in the music conditions, but they tended to show greater trust behavior and rated faces and nicknames as more trustworthy compared to those in the no-music conditions, although the trends did not reach statistical significance.

All studies on choral singing reported that participants had experienced social bonding, and OXT levels declined in most cases Bowling et al., (2022); Fancourt et al., (2016); Keeler et al., (2015), except in improvised choral singing, where OXT levels increased Keeler et al., (2015).

Last, participants frequently reported subjective improvements in relaxation and mood scales after musical activities across studies. However, these changes were not always accompanied by increases in peripheral OXT levels Bowling et al., (2022); Eerola et al., (2021); Fancourt et al., (2016); Good and Russo, (2022); Grape et al., (2003); Kreutz, (2014); Maramis et al., (2021); Nilsson, (2009); Wulff et al., (2021).

Overall, improvements in psychosocial outcomes do not correspond with changes in peripheral OXT levels, and consistent OXT response patterns were observed only for specific psychosocial outcomes.

### 3.4 Effects of contextual factors on patterns of OXT responses and psychosocial outcomes

#### 3.4.1 Patterns of OXT responses

Analyses of OXT responses across studies consistently revealed interaction effects, which indicated that patterns of OXT change were context-dependent (see Table 7). Kreutz (2014) demonstrated a significant time × condition interaction for salivary OXT responses (*F*(1,21) = 7.988, *p* < 0.05). The dependence on activity was further supported by several studies: Filippa et al. (2021) reported a significant time × condition interaction (χ^2^(2) = 6.99, *p* = 0.03), as did Good and Russo (2022) (*z* = −2.142, *p* = 0.032). Schladt et al. (2017) observed a significant time × context interaction (*F*(4) = 7.27, *p* < 0.001), and Bowling et al. (2022) found a significant time × vocal mode interaction (χ^2^(1) = 5.7, *p* = 0.018). Evidence for OXT's sensitivity to stress signals was provided by Ooishi et al. (2017), who observed a significant time × tempo interaction (*F*(1,22) = 13.44, *p* = 0.0014). However, Bowling et al. (2022) found no significant time × social context interaction (*p* = 0.230) or time × vocal mode × social context interaction (*p* = 0.190), indicating that OXT responses were not significantly influenced by the explicitly manipulated social setting in their study.

**Table 7:** Effects of contextual factors on OXT response patterns and psychosocial outcomes.

**Author (year)**	**Outcome**	**Statistical analysis**		**Contextual factor**
		**Interaction**	**Result**	
**Physiological outcome**				
Bowling et al. (2022)	Salivary OXT levels	Time × vocal mode	χ^2^(1) = 5.7, *p* = 0.018	Activity (singing vs. speaking)
Bowling et al. (2022)	Salivary OXT levels	Time × social context	*p* = 0.230 (non-significant)	Social cue (together vs. alone)
Bowling et al. (2022)	Salivary OXT levels	Time × vocal mode × social context	*p* = 0.190 (non-significant)	Activity (singing vs. speaking); Social cue (together vs. alone)
Filippa et al. (2021)	Salivary OXT levels	Time × condition	χ^2^(2) = 6.99, *p* = 0.03	Activity (maternal singing vs. maternal speaking vs. standard care)
Good and Russo (2022)	Salivary OXT levels	Time × condition	*z* = −2.142, *p* = 0.032	Activity (choral singing vs. solo singing)
Kreutz (2014)	Salivary OXT levels	Time × condition	*F*(1,21) = 7.988, *p* < 0.05	Activity (choral singing vs. chatting)
Ooishi et al. (2017)	Salivary OXT levels	Time × tempo	*F*(1,22) = 13.44, *p* = 0.0014	Stress signal (slow music vs. fast music)
Schladt et al. (2017)	Salivary OXT levels	Time × context	*F*(4) = 7.27, *p* < 0.001	Activity (choral singing vs. solo singing)
**Psychosocial outcome**				
Bowling et al. (2022)	IOS scale	Time × vocal mode	χ^2^(1) = 7.06, *p* = 0.008	Activity (singing together vs. speaking together)
Bowling et al. (2022)	PANAS	Time × vocal mode × social context	χ^2^(1) = 10.54, *p* = 0.001	Activity (singing vs. speaking); Social cue (together vs. alone)
Good and Russo (2022)	PANAS	Time × condition	*z* = −2.321, *p* = 0.020	Activity (choral singing vs. solo singing)
Jezova et al. (2013)	STAI-S	Time × condition	*F*(1, 26) = 5.10, *p* < 0.05	Stress signal (unpleasant music vs. pleasant music)
Kreutz (2014)	*Ad hoc* questionnaire of subjective feelings-positive feelings	Time × condition	*F*(1,20) = 9.655, *p* < 0.01	Activity (choral singing vs. chatting)
Schladt et al. (2017)	STADI-S-excitement	Time × context	*F*(1) = 6.51, *p* = 0.015	Activity (choral singing vs. solo singing)
Schladt et al. (2017)	STADI-S-happiness	Time × context	*F*(1) = 5.27, *p* = 0.028	Activity (choral singing vs. solo singing)

This table presents studies that detected significant statistical interactions, which indicates that the temporal patterns of OXT levels or psychosocial outcomes differed across contexts. The “Result” column shows test statistics and p-values for the interaction. All significance is defined as *p* < 0.05.

IOS, Inclusion of Other in the Self; OXT, oxytocin; PANAS, Positive and Negative Affect Schedule; STADI-S, the state subscale of State-Trait Anxiety-Depression Inventory; STAI-S, the state subscale of State-Trait Anxiety Inventory.

#### 3.4.2 Patterns of psychosocial outcomes

Psychosocial outcomes exhibited varying degrees of context-dependence (see Table 7). Kreutz (2014) found a significant time × condition interaction for positive feelings (*F*(1,20) = 9.655, *p* < 0.01). Schladt et al. (2017) observed significant time × context interactions for excitement (*F*(1) = 6.51, *p* = 0.015) and happiness (*F*(1) = 5.27, *p* = 0.028), but not for worry or sadness. Jezova et al. (2013) examined the effects of pleasant vs. unpleasant music and found a significant time × condition interaction for state anxiety (*F*(1, 26) = 5.10, *p* < 0.05). In Bowling et al. (2022), the social bonding measure (IOS scale) showed a significant time × vocal mode interaction (χ^2^(1) = 7.06, *p* = 0.008). In contrast, the subjective emotion measure (PANAS) in the same study exhibited a significant three-way interaction among time, vocal mode, and social context (χ^2^(1) = 10.54, *p* = 0.001). The findings indicate that changes in psychosocial outcomes, particularly positive affect measures, were significantly influenced by activity and social cues.

### 3.5 The characteristics of OXT measurements

All the included studies used peripheral OXT measurements as biomarkers, and they varied in collection protocols, sample types, and quantification methods, as summarized in Table 8. Twelve studies provided details on the time of sample collection. Of these, nine studies scheduled collection during the afternoon or evening hours to avoid interference from the cortisol awakening response (CAR). The remaining studies adopted different protocols: one study collected samples across many time points throughout the day (morning, afternoon, and evening), and another between 9:00 and 13:00. Notably, Maramis et al. (2021) was the sole study that conducted sample collection during the CAR period in the early morning.

**Table 8:** The characteristics of OXT measurements.

**Author (year)**	**Intervention duration**	**Sample type**	**Collection time**	**Measurement time point**	**Extraction**	**Quantification**	**OXT level (pg/ml)**
Bowling et al. (2022)	20 min	Saliva	18:00–20:00; 10 min/sample	*t* = −5 (−10–0), 30 (25–35) min	Yes	EIA	45.9–305.5^a^
Corbett et al. (2011)	120 min (3-month program)	Plasma	Unclear	Before and after the entire program	Unclear	EIA	N/A
Dai et al. (2012)	Music: 5–8 min, Cold water: < 45 s	Serum	13:00–16:00	Baseline: *t* = −5, 0 min, Experiment: *t* = 1, 5, 10, 15, 20, 25, 30, 45 min	No	EIA	WS: 130–7500; TC: 80–300
Eerola et al. (2021)	20 min	Plasma	Unclear	Baseline: *t* = 0 min, Experiment 1: *t* = 20 min, Experiment 2: *t* = 40 min	Yes	EIA	0.59–25.58
Fancourt et al. (2016)	70 min	Saliva	19:00–20:15	*t* = 0, 75 min	No	Fluorescence bead-based multiplex immunoassay	3.62–5.15
Filippa et al. (2021)	10 min	Saliva	Unclear	*t* = 0, 10 min	Not required	RIA	0.78–1.4
Filippa et al. (2023)	10 min	Saliva	Unclear	*t* = 0, 10 min	Not required	RIA	2.6–3.0
Good and Russo (2022)	45 min	Saliva	At the same time of day (evening)	*t* = 0, 45 min	No	Electrochemiluminescence assay	7.8–14.8
Grape et al. (2003)	45 min	Serum	9:00–20:15	Before and 30 min after the lesson	No	EIA	469–788
Jezova et al. (2013)	45 min	Plasma	12:15–16:00	*t* = 0, 15, 30, 45, 60, 90 min	Not required	RIA	N/A
Keeler et al. (2015)	Nearly 6 min	Plasma	Unclear	*t* = −5, 6 min	No	EIA	90–370
Kreutz (2014)	30 min	Saliva	Unclear	*t* = 0, 30 min	Unclear	EIA	13.04–18.08
Maramis et al. (2021)	15 min (8-week program)	Saliva	In the morning	Before and after the entire program	No	EIA	82.3–173^b^
Nilsson (2009)	30 min	Serum	12:00–13:00	*t* = 0, 30, 60 min	Yes	EIA	62.6–73.9
Ooishi et al. (2017)	20 min	Saliva	14:00–18:00; 1–3 min/sample	*t* = 0, 20 min	Yes	EIA	2.26–17.02
Palumbo et al. (2022)	45 min (6-week program)	Serum	Unclear	Before and after the entire program	No	EIA	59.26–68.73
Riedl et al. (2017)	During 40 rounds of the trust game	Plasma	9:00–13:00	Pre-game, post 10 rounds, post 20 rounds, post 30 rounds, post 40 rounds, 10 min after the game	Yes	Fluorescence immunoassay	27–51
Schladt et al. (2017)	20 min	Saliva	18:00–20:30; 1 min/sample	Baseline: *t* = −20, 0 min, Experiment: *t* = 10, 20 min, Post-session: *t* = 40 min	Not required	RIA	0–13
Wulff et al. (2021)	30 min	Saliva	13:00–16:00	*t* = 0, 30 min	Not required	RIA	1.00–1.11
Yuhi et al. (2017)	Recital: 5–60 min, Practice: 80–155 min, Free condition: 90–200 min	Saliva	2–4 min/sample	10 min before and 10 min after each session	No	EIA	83–265^a^

For OXT levels, the values in the table indicate the range described in the references or estimated from charts or their supplementary data. The starting time points of interventions were corrected to 0 min.

EIA, enzyme immunoassay; RIA, radioimmunoassay; TC, typical control; WS, Williams syndrome.

^a^High concentrations are likely due to prolonged collection.

^b^The quantitative unit was not reported.

In short-term interventions, samples were often collected immediately before and after a single session to measure acute OXT changes. Six studies collected samples at three or more time points (Dai et al., 2012; Eerola et al., 2021; Jezova et al., 2013; Nilsson, 2009; Riedl et al., 2017; Schladt et al., 2017), whereas the others relied on pre-test and post-test measurements. Long-term interventions assessed baseline OXT levels before and after the entire program, which lasted from several weeks to months. However, long-term interventions generally showed no significant changes in baseline OXT levels.

Sample types included plasma (*n* = 5), serum (*n* = 4), and saliva (*n* = 11). Generally, plasma and serum samples showed higher OXT concentrations (ranging from tens to hundreds of pg/ml) compared to saliva samples (approximately 0–20 pg/ml). In terms of quantification methods, 12 studies used enzyme immunoassay (EIA), of which four applied sample extraction procedures prior to EIA. Five studies used radioimmunoassay (RIA), one study used electrochemiluminescence assay, and two used fluorescence immunoassay. Studies that omitted sample extraction before EIA tended to report higher OXT concentrations (Dai et al., 2012; Grape et al., 2003; Keeler et al., 2015; Maramis et al., 2021; Palumbo et al., 2022; Yuhi et al., 2017) than those that included extraction steps (Bowling et al., 2022; Eerola et al., 2021; Nilsson, 2009; Ooishi et al., 2017).

Therefore, methodological differences in OXT measurements may contribute to inconsistencies in OXT levels reported across studies.

## 4 Discussion

### 4.1 The bidirectional modulation of OXT

#### 4.1.1 Context-dependence of OXT responses

High-quality studies have shown that music can lead to both increases and decreases in peripheral OXT levels. Overall, clinical condition appears to be the most influential factor in modulating OXT release. For instance, individuals with Williams syndrome exhibit exaggerated OXT release in response to their favorite positive music, suggesting dysregulation of the OXTergic system (Dai et al., 2012). In pregnant and postpartum mothers, the OXTergic system is highly active yet peripheral OXT levels tend to be low (Filippa et al., 2023; Wulff et al., 2021), since pregnancy inhibits OXT secretion but enhances OXT receptor (OXTR) expression and enzymatic degradation (Uvnas-Moberg, 2024).

**Figure 2:**
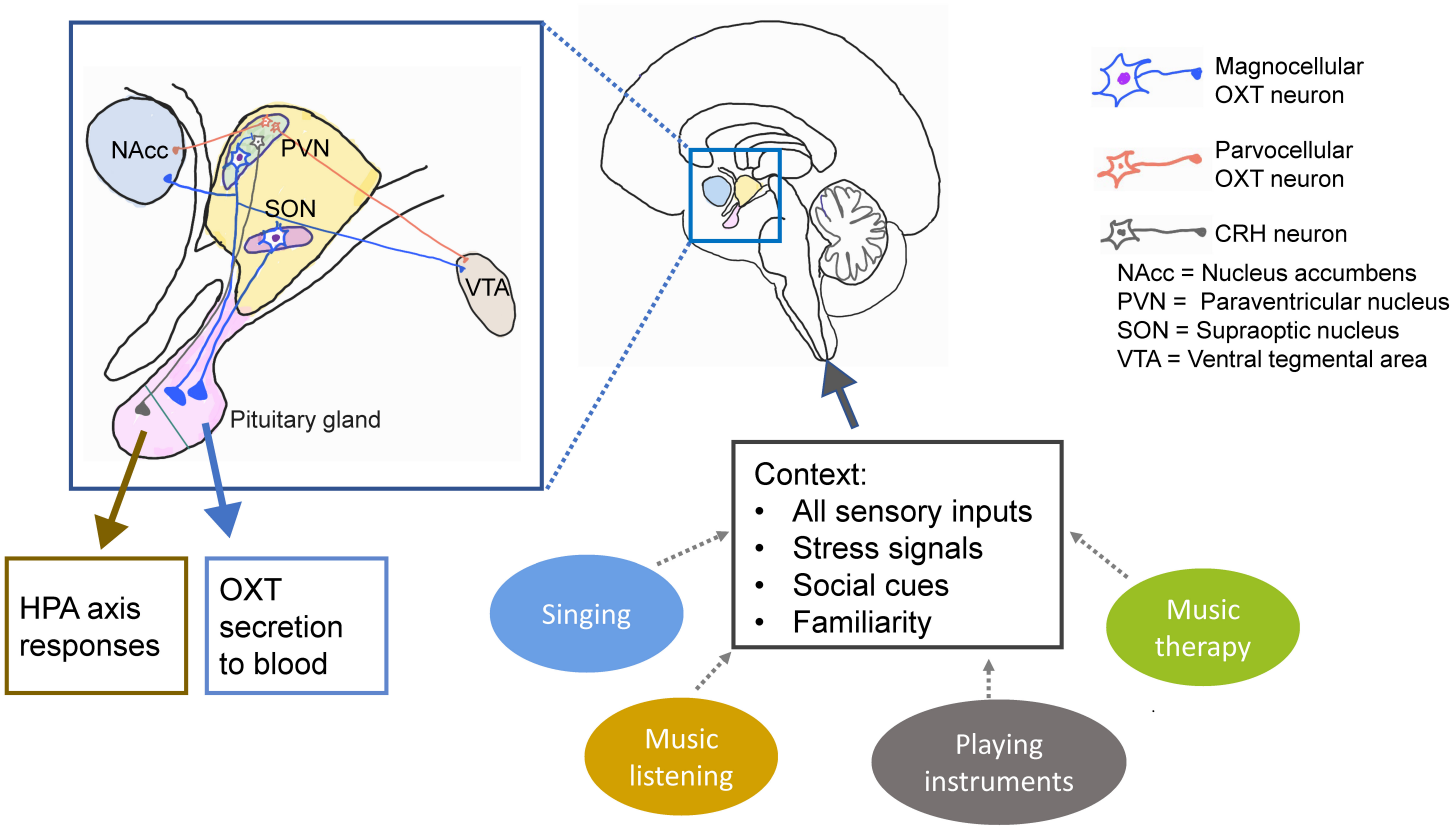
Music shapes OXT response via context-dependence.

Context-dependence can explain the variability in OXT responses across studies. Under basal conditions, central and peripheral OXT levels show no significant correlation (Kagerbauer et al., 2013; Valstad et al., 2017), since peripheral OXT reflects only the activity of magnocellular OXT neurons projecting to the posterior pituitary but central release involves both magnocellular and parvocellular OXT neurons (Bowling, 2023). However, during acute stress or strong physiological or social stimulation, coordinated OXT release in the CNS and periphery may occur and produce higher correlations between central and peripheral OXT levels (Martin et al., 2018; Valstad et al., 2017). As illustrated in Figure 2, music can induce such release by shaping the context, which is comprised of several elements, including the type of activity, stress signals, social cues, and familiarity with the music or social setting. The auditory signals travel through the classical pathway to the auditory cortex, while contextual inputs, including all sensory inputs, stress signals, and social cues, go through the non-classical pathway to the limbic system and the hypothalamus. The inputs are integrated in the hypothalamus, which may trigger coordinated or independent release of OXT in the CNS and periphery, and activate or deactivate the hypothalamic-pituitary-adrenal (HPA) axis.

Nevertheless, the dimensions that constitute the context differ in their importance. Our contextual analysis (Tables 6, 7) indicates that activity and stress signals have combined effects on OXT responses, with qualitative analysis showing that activity is the primary driver, while stress signals may reverse these effects under certain conditions. Social cues and familiarity showed limited predictive power. Statistical evidence also supports the patterns. Schladt et al. (2017) found a significant time × context interaction, showing that OXT changes over time depend on contexts, consistent with other studies (Filippa et al., 2021; Good and Russo, 2022; Kreutz, 2014). Bowling et al. (2022) distinguished two contextual factors, vocal mode and social context, and identified vocal mode (corresponding to activity type) as the key factor that influences OXT response patterns, with social context showing no significant effect. Ooishi et al. (2017) confirmed OXT's responsiveness to stress signals, as evidenced by a significant time × tempo interaction effect in a music-listening task that manipulated arousal or stress levels via tempo.

As for the weak effect of the social context, we attribute this to potential issues in the current experimental paradigm. Just as HPA axis activation requires high-stress conditions, OXT modulation may need sufficiently demanding social cues. Keeler et al. (2015) observed the influence of social cues through improvised group singing, which involved trust, synchrony, and real-time interaction. Instead, the structured singing tasks in Schladt et al. (2017) and Bowling et al. (2022) required participants to focus on vocal performance with limited spontaneous social interactions and should have been insufficient to reveal the effects of social cues. As a result, the context-dependence of OXT responses to music is specifically attributed to its reliance on two factors, namely the activity type and the stress signals, based on the statistical findings.

#### 4.1.2 Functional adaptation of OXT to musical contexts

OXT exhibits temporally and spatially specific release patterns, and peripheral release can occur independently of or in parallel with central release (Jurek and Neumann, 2018). In the circulation, OXT follows a pulsatile release pattern, and pulse characteristics are strongly linked with baseline socio-emotional functioning. Mean OXT pulse height and pulse mass were negatively correlated with avoidant attachment and positively correlated with perceived social support (Baskaran et al., 2017). It suggested that temporal dynamics of peripheral OXT release carry crucial psychosocial information.

In the CNS, spatial specificity of OXT release allows for nuanced modulation. OXT activates OXTRs, which are coupled with Gq or Gi/o proteins, resulting in diverse downstream effects (Jurek and Neumann, 2018). For example, OXT release directly activates dopaminergic neurons and indirectly inhibits them via local GABAergic interneurons, but the relative magnitudes of the two mechanisms differ in the ventral tegmental area (VTA) and substantia nigra pars compacta (SNc; Xiao et al., 2017). OXT functions as a allosteric modulator of μ opioid receptor (MOR), and enhances the MOR signaling without altering receptor affinity (Meguro et al., 2018). In the striatum, OXTR activation on astrocytes inhibits glutamate release (Amato et al., 2022), and D2R-OXTR heterocomplexes facilitate dopaminergic signaling (Hernandez-Mondragon et al., 2023; Pérez de la Mora et al., 2016). The differential modulation of OXT supports its excitatory and inhibitory effects, which depend on cell type, receptor coupling, and local neurotransmitter interactions.

Thus, OXT increases or decreases play different roles in reward processing, social cognition, and social behaviors:

(1) Reward processing: the ventral striatum is central in reward processing. The nucleus accumbens (NAcc) is responsible for recognizing the salience and valence of stimuli (Cooper and Knutson, 2008). The left NAcc recognizes pleasure, while the right NAcc responds to all noteworthy stimuli whether they are positive or negative. When OXT increases, it may promote dopaminergic signaling (Xiao et al., 2017) and enhance the output of the striatum.
(2) Empathy network: the ventromedial prefrontal cortex (vmPFC), a core area of the empathy network (Taruffi et al., 2021), normally receives inhibitory OXT projections. Increased OXT reduces activity in vmPFC, accelerates the response to social cues, reduces recall accuracy, and blurs the boundaries between self and others (Zhao et al., 2016). Decreased OXT might enhance the local activity of the empathy network and right striatum, strengthen emotional responses, and promote deeper emotional engagement with music, though direct evidence for this pattern in musical contexts remains to be established.
(3) Social behavior: in both approach and avoidance behaviors, increased OXT reduces right striatum activity, potentially suppressing excessive emotional interference and promoting appropriate behavioral selection. Although OXT may weaken overall motivation, it improves the accuracy of approach to positive stimuli (Yao et al., 2018). This effect may reflect a compensatory mechanism in which motivation control shifts toward the left striatum when the right striatal activity is reduced.

The bidirectional modulation of OXT suggests that the OXTergic system adapts flexibly to musical context. Happy music primarily activates the left NAcc (Trost et al., 2012), and OXT release may facilitate dopaminergic signaling (Dai et al., 2012). Sad music selectively activates the right NAcc over the left (Trost et al., 2012), and is often accompanied by peripheral OXT decreases, especially in females with high trait empathy (Eerola et al., 2021).

Furthermore, there is a bidirectional relationship between the OXTergic system and the HPA axis (Bowling, 2023; Harvey, 2020; Jurek and Neumann, 2018). Under acute stress conditions, three temporal patterns may occur:

(1) Concurrent release of OXT and adrenocorticotropic hormone (ACTH)/cortisol (Keeler et al., 2015);
(2) An initial OXT increase that attenuates HPA activation (Jezova et al., 2013; Keeler et al., 2015);
(3) HPA activation that selectively inhibits OXT release from the PVN (Jezova et al., 2013; Keeler et al., 2015).

Engaging in low-exertion social activities under low-stress conditions is conducive to OXT release since OXT is primarily involved in physiological relaxation rather than psychological relaxation (Ooishi et al., 2017). Slow-tempo music is more likely to induce OXT release without inhibition by the HPA axis (Ooishi et al., 2017; Wulff et al., 2021), as it is usually perceived as more pleasant and relaxing (Ooishi et al., 2017). In contrast, fast-tempo music or singing may initially activate the HPA axis due to increased arousal or stress, which can temporarily suppress OXT release, even though the HPA responses may decrease by the end of the intervention (Bowling et al., 2022; Fancourt et al., 2016; Good and Russo, 2022; Grape et al., 2003; Jezova et al., 2013; Keeler et al., 2015; Ooishi et al., 2017; Schladt et al., 2017). Additionally, prolonged singing sessions (e.g., over an hour) may lead to fatigue and limit other social interactions (Fancourt et al., 2016). If the reward from music is insufficient to counteract the earlier inhibition, OXT levels may remain unchanged or even decrease, as observed in studies where the participants sang touching sublime religious songs (Bowling et al., 2022; Schladt et al., 2017).

In summary, the findings challenge the assumption that higher OXT levels are beneficial. Instead, both increases and decreases in OXT levels may support adaptive emotional and social functions. Importantly, the context-dependence of music and OXT emphasizes the need to consider baseline physiological and psychological states, intervention types, and environmental factors.

### 4.2 Peripheral OXT and subjective outcomes

Discrepancies between peripheral OXT levels and psychosocial outcomes have led to ongoing debate about the relationship between central and peripheral OXT activity. While context-dependence may account for the variability and direction of peripheral OXT responses, subjective outcomes exhibit different context-dependence.

Evidence from Bowling et al. (2022) implied the dissociation. The patterns of OXT responses were mainly determined by vocal mode (time × vocal mode interaction) rather than social cues. IOS scale also showed a significant time × vocal mode interaction, which suggested that social bonding might be associated with the physiological processes behind vocal synchrony. However, PANAS showed a significant three-way interaction (time × vocal mode × social context), indicating that the changes of affective experiences were dependent on both the activity and social cues. In addition, there were no significant correlations between the pre-post change of OXT and self-reported emotion, mood or stress scores (Fancourt et al., 2016; Ooishi et al., 2017). Thus, different outcome measures are sensitive to different contextual factors. OXT responses should be regarded as intrinsic physiological adaptations to musical activities. Since OXT secretory dynamics (e.g., pulse characteristics) are more likely to reflect subjective socio-emotional functioning than a single measurement or pre-post change (Baskaran et al., 2017), pre-post changes of OXT often fail to predict subjective outcomes, which are heavily influenced by placebo effects, expectations, and the infeasibility of blinding in music-based interventions. As a result, perceived benefits may reflect cognitive and cultural interpretations of musical meaning.

Behavioral changes following musical activities are likely mediated by central mechanisms not fully captured by peripheral OXT measurements (Martins et al., 2020). For example, social cognition and reward processing can be mediated by OXT and OXTR, while social communication relies more on arginine-vasopressin (AVP) and its receptor (V1aR; Song and Albers, 2018). Since OXT and AVP share structural similarities and can activate each other's receptors, this phenomenon, “cross-talk” (Song and Albers, 2018), is likely to participate in shaping social behaviors in musical contexts. Therefore, peripheral OXT levels have limited predictive value for subjective outcomes. When interpreting outcomes regarding social cognition, social reward, or social behavior, we recommend considering the OXT-AVP systems that mediate the adaptive responses to social stimuli.

### 4.3 Clinical implications

The bidirectional modulation of OXT suggests that therapeutic strategies need to move beyond merely increasing OXT levels, as OXT enhances the salience of both positive and negative social cues, encouraging social approach in safe contexts but inducing avoidance in aversive environments (Steinman et al., 2019). From a translational medicine perspective, the context-dependence of OXT responses implies that existing music-based interventions could be refined by considering the activity type and stress signals involved.

For social anxiety disorder (SAD) patients, social cues belong to intense stress signals since they usually interpret social cues negatively and lack motivation for positive social interaction. Based on our contextual analysis, at the initial stage, treatment should begin with a safe setting using neutral, calming, or peaceful music, with limited social interactions. It is advisable that the musical emotions not completely simulate anxiety to avoid overwhelming negative feelings in patients. Social engagement can be gradually introduced only after basic trust is established and the emotional state is stabilized. We recommend structured group singing in a small choir (Keeler et al., 2015) for SAD patients, as it provides appropriately limited social cues which is sufficient for therapeutic effects but not overwhelming.

Some typical characteristics of ASD, such as deficits in imitation, emotional empathy, and attributing intentions to others, reflect the mirror system dysfunction (Cattaneo and Rizzolatti, 2009). For ASD children, rhythmic musical activities could mobilize the mirror systems, improve self-control, and help process acoustic prosody for conversational quality (Wynn et al., 2022). Because rhythmic music involves precise timing prediction and synchronization, patients need to pay more attention to social cues. Patients who are sensitive to OXT will benefit from improvement in social information processing. Thus, group drumming (Yuhi et al., 2017) and musical theater therapy (Corbett et al., 2011) are recommended for individuals with ASD. The therapy design requires setting up lessons of different levels and breaking them down into manageable parts to learn. Rhythm complexity needs to increase gradually, from simple, accessible patterns to more complex synchronization tasks, to avoid frustrations for beginners.

Understanding the mechanisms through which music influences emotion regulation and social bonding benefits the design of music therapy, as it enables a better understanding of which strategies are more effective in mobilizing the neural networks to achieve therapeutic goals.

### 4.4 Strengths and limitations of the included study

Several included studies demonstrated methodological rigor that strengthens confidence in their findings. The RCTs provided stronger evidence for causal relationships between music-based interventions and OXT changes. Some studies accounted for biological and environmental confounders, used validated OXT assays with extraction procedures (Gan et al., 2023), and employed real-world settings that enhanced ecological validity. An advantage was the predominant use of saliva samples. Unlike plasma OXT, which fluctuates rapidly due to its 7-min half-life (Shafer et al., 2025) and pulsatile release pattern (Baskaran et al., 2017), salivary OXT can provide more stable measurements since it has a longer half-life (Quintana et al., 2018).

However, the studies exhibit several limitations that necessitate discussion. A primary concern is the reliance on pre-post measurements of peripheral OXT, which may not reflect central OXT activity (Kagerbauer et al., 2013; Valstad et al., 2017) or be correlated with attachment style, perceived social support, and emotional awareness (Baskaran et al., 2017). Timing issues were also evident in some studies. Specifically, OXT's dramatic fluctuations within CAR period could potentially mask intervention effects. Comparability across studies was hindered by inconsistencies in sampling methods and quantification techniques (Lefevre et al., 2017), as well as insufficient reporting of musical and contextual details. Moreover, common issues like small sample sizes and the lack of negative controls reduced the internal validity. While fasting and exercise restrictions did not have significant influence on OXT levels (Engel et al., 2019), other behavioral confounders, such as hospital anxiety or sexual activity prior to sampling, still need to be considered (Jurek and Neumann, 2018).

### 4.5 Strengths and limitations of the current study

This review addressed the research questions through comprehensive search strategies, predefined eligibility criteria, and structured syntheses that integrated heterogeneous findings. Our contextual analysis provides a key insight by identifying four distinct contextual dimensions and their different contribution on OXT modulation.

However, there were several limitations in the review. First, it was mainly conducted by one reviewer, which may introduce selection bias, and restrictions to English publications could have excluded relevant studies. None of the included studies examined OXTR gene polymorphisms, which associate with social behavior variability in clinical populations (Harrison et al., 2015) and may influence individual responses to music. Additionally, no studies directly investigated receptor interactions such as functional complexes of OXTR with dopamine, serotonin, and opioid receptors. Only Fancourt et al. (2016) reported correlated declines of OXT and beta-endorphin after choral singing. Thus, the interpretation scope of the review is limited.

## 5 Conclusion

The context-dependence of OXT responses to music has significant implications for clinical practice and research. Evidence supports a bidirectional relationship in which music induces subtle emotional responses, which are further modulated by OXT. The effectiveness of music-based interventions may not rely on the direction of OXT change but on the adaptive modulation of OXT. For clinical applications, music-based interventions should be carefully tailored to specific populations and contexts. Larger sample sizes, standardized outcome measures, and well-controlled experimental designs are needed in the future. Given the limitations of peripheral OXT measurements, we suggest that future studies explore central OXT signaling or receptor interactions. Integrating genetic studies and peptide assessments, the use of OXTR agonists or antagonists, combined with neuroimaging techniques such as functional magnetic resonance imaging (fMRI), may provide deeper insights into how music modulates brain activity through the OXTergic system.

## Data Availability

The original contributions presented in the study are included in the article/Supplementary material, further inquiries can be directed to the corresponding author.

## Supplementary material

Supplementary material S1 Systematic review protocol.

Supplementary material S2 Peripheral OXT changes induced by music over time.
