# Supplementary material for: Music's context-dependent influence on oxytocin, social bonding, and emotion regulation: a systematic review
Source: Front Cognit. 2026 Jan 2;4:1678665. doi: 10.3389/fcogn.2025.1678665 (PMC13281211; doi:10.3389/fcogn.2025.1678665)
Supplement: Supplementary material S1 — Systematic review protocol. [file Data_Sheet_1.pdf]

frontiers

Systematic Review Protocol

1 Background

1.1 Rationale

The mechanisms through which music influences mental health are linked to oxytocin (OXT) (Bowling, 2023), which is an important neuropeptide that modulates social behaviors and emotional responses (Jurek and Neumann, 2018; Song and Albers, 2018). Studies have suggested that music could influence OXT levels, improving musical experiences and social bonding (Bowling, 2023; Good and Russo, 2022; Keeler et al., 2015). However, music therapy experiments are often criticized for lacking rigorous designs, standardized procedures, and consistent measurements. The methodologies, including music-based interventions, study designs, OXT measurement methods, populations, and other outcome measures, vary widely in this field (Engel et al., 2019; Tabak et al., 2023), making it difficult to compare the results of the studies. Therefore, a systematic review is essential to evaluate the evidence of music therapy and OXT, with a view to figuring out the patterns between different studies, conceptualizing the mechanisms, and suggesting directions for future research.

1.2 Objectives

Primary Research Question: How do individual and group musical activities influence mental health through the OXTergic system in terms of social bonding and emotion regulation?
Secondary Research Questions:
a) How do OXT levels change in different populations (categorized by age, sex, clinical conditions, and baseline OXT levels) in music-based interventions?
b) Which characteristics of music-based interventions (e.g., activity type, duration, frequency, genre, tempo, music form, context) can be associated with significant changes in OXT levels and psychosocial outcomes?
c) What are the strengths and limitations of OXT measurements in music-based interventions?

2 Methods

2.1 Information Sources and Search Strategy

Databases: PubMed, Embase, Scopus, Web of Science Core Collection, and PsycInfo (EBSCOhost). Search terms will include a variety of terms related to music therapy, OXT, social bonding, and emotion regulation. The search strategy will be tailored for each database.

Sample Search String:
("Music Therapy" OR "Music" OR "Music intervention" OR "music-based interventions" OR "sing" OR "singing" OR "singer" OR "songs" OR "listen" OR "listening" OR "listener" OR "rhythm" OR "Rhythmic Auditory Stimulation" OR "RAS" OR "religion" OR "religious" OR "ritual" OR "spiritual" OR "ceremony" OR "shared grief" OR "communal mourning" OR "collective grief" OR "collective mourning" OR "sharing of emotions" OR "emotional sharing" OR "affect-sharing" OR "Affective sharing")

AND ("Oxytocin" OR "OXT" OR "oxytocinergic" OR "Oxytocin receptor" OR "OXTR" OR "OTR")
AND ("social behavio*" OR "social" OR "bond*" OR "prosocial" OR "attachment" OR "engag*" OR "interact*" OR "trust" OR "empath*" OR "affiliat*" OR "affect regulation" OR "emotion*" OR "mood" OR "anxiety" OR "depression" OR "stress" OR "pain*" OR "relax*")

2.2 Eligibility Criteria

Inclusion Criteria:
a) Study design: Randomized controlled trials (RCTs), quasi-experiments, observational studies
b) Publication date: Up to June 2024
c) Language: English
d) Population: All age groups of healthy populations or any clinical populations
e) Intervention: Music therapy or musical activities, including listening to music, singing, playing instruments, whether in group or individually.
f) Outcome: Studies must include data on OXT measurements. In addition to OXT, studies need to report any positive or adverse health-based outcome, any objective health-based clinical outcome measure, or any subjective outcome.

Exclusion Criteria:
a) Non-human studies
b) Reviews or editorials without original data
c) Studies simply focusing on drug interventions (but studies comparing music therapy with drug therapy will be included)
d) Book chapters or conference papers
e) Non-peer-reviewed papers

2.3 Study Selection

One reviewer will screen titles and abstracts, followed by a full-text review of potentially eligible studies. In cases of uncertainty, the reviewer will consult with the second reviewer to reach a decision.

2.4 Data Extraction

One reviewer will use a standardized form to extract data and double-check it to minimize errors. The extracted information is listed as follows:
a) General information: title, author, publication year, journal
b) Study characteristics: study design, sample size, population, type of intervention, type of outcome
c) Participant: recruitment, background, eligibility criteria, age, sex, ethnicity, clinical condition, baseline imbalance, follow-up, withdrawal
d) Intervention and comparison: group name, number of participants, setting, procedure, frequency, duration
e) Outcome: outcome name, definition, time point, assessment tool
f) Results
g) Findings
h) Statistical analysis

2.5 Quality Assessment

One reviewer will use JBI checklists to assess the study quality, depending on the study design.

2.6 Data Analysis and Synthesis

2.6.1 Framework Synthesis

One reviewer will use a theoretical framework to guide data analysis and synthesis. The framework synthesis was conducted through the five-step process (Brunton et al., 2020):
a) Familiarization: Conducting scoping searches and thoroughly engaging with sources.
b) Framework Selection: Selecting an appropriate framework based on the potential to address the research questions.
c) Indexing: Extracting and coding the data from the included studies. It involves identifying and categorizing relevant data according to framework components.
d) Charting: The extracted data was organized into tables or charts to help analyze, compare, and identify patterns, themes, and relationships.
e) Mapping and Interpretation: Synthesizing the charted data, identifying connections between themes, and explaining the findings.

2.6.2 Narrative Synthesis

One reviewer will conduct a narrative synthesis to complement framework synthesis and provide contextual information. The narrative synthesis involves (Impellizzeri and Bizzini, 2012):
a) Preliminary Synthesis: Summarizing findings within each outcome domain.
b) Exploration of Relationships: Identifying trends, contradictions, and other factors.
c) Robustness Assessment: Considering methodological limitations that may influence the interpretations.

3 References

Bowling, D.L. (2023). Biological principles for music and mental health. *Translational Psychiatry* 13, 374. doi: 10.1038/s41398-023-02671-4.

Brunton, G., Oliver, S., and Thomas, J. (2020). Innovations in framework synthesis as a systematic review method. *Research Synthesis Methods* 11(3)**,** 316–330. doi: 10.1002/jrsm.1399.

Engel, S., Laufer, S., Miller, R., Niemeyer, H., Knaevelsrud, C., and Schumacher, S. (2019). Demographic, sampling- and assay-related confounders of endogenous oxytocin concentrations: a systematic review and meta-analysis. *Frontiers in Neuroendocrinology* 54, 100775. doi: 10.1016/j.yfrne.2019.100775.

Good, A., and Russo, F.A. (2022). Changes in mood, oxytocin, and cortisol following group and individual singing: a pilot study. *Psychology of Music* 50(4)**,** 1340–1347. doi: 10.1177/03057356211042668.

Impellizzeri, F.M., and Bizzini, M. (2012). Systematic review and meta-analysis: a primer. *International Journal of Sports Physical Therapy* 7(5)**,** 493–503. https://pmc.ncbi.nlm.nih.gov/articles/PMC3474302/

Jurek, B., and Neumann, I.D. (2018). The oxytocin receptor: from intracellular signaling to behavior. *Physiological Reviews* 98(3)**,** 1805–1908. doi: 10.1152/physrev.00031.2017.

Keeler, J.R., Roth, E.A., Neuser, B.L., Spitsbergen, J.M., Waters, D.J.M., and Vianney, J.M. (2015). The neurochemistry and social flow of singing: bonding and oxytocin. *Frontiers in Human Neuroscience* 9, 518. doi: 10.3389/fnhum.2015.00518.

Song, Z., and Albers, H.E. (2018). Cross-talk among oxytocin and arginine-vasopressin receptors: Relevance for basic and clinical studies of the brain and periphery. *Frontiers in Neuroendocrinology* 51**,** 14–24. doi: 10.1016/j.yfrne.2017.10.004.

Tabak, B.A., Leng, G., Szeto, A., Parker, K.J., Verbalis, J.G., Ziegler, T.E., et al. (2023). Advances in human oxytocin measurement: challenges and proposed solutions. *Molecular Psychiatry* 28(1)**,** 127–140. doi: 10.1038/s41380-022-01719-z.
