# Supplementary material for: Music's context-dependent influence on oxytocin, social bonding, and emotion regulation: a systematic review
Source: Front Cognit. 2026 Jan 2;4:1678665. doi: 10.3389/fcogn.2025.1678665 (PMC13281211; doi:10.3389/fcogn.2025.1678665)
Supplement: Supplementary material S2 — Peripheral OXT changes induced by music over time. [file Data_Sheet_2.pdf]

Supplementary Material

1 Supplementary Figures

(A)

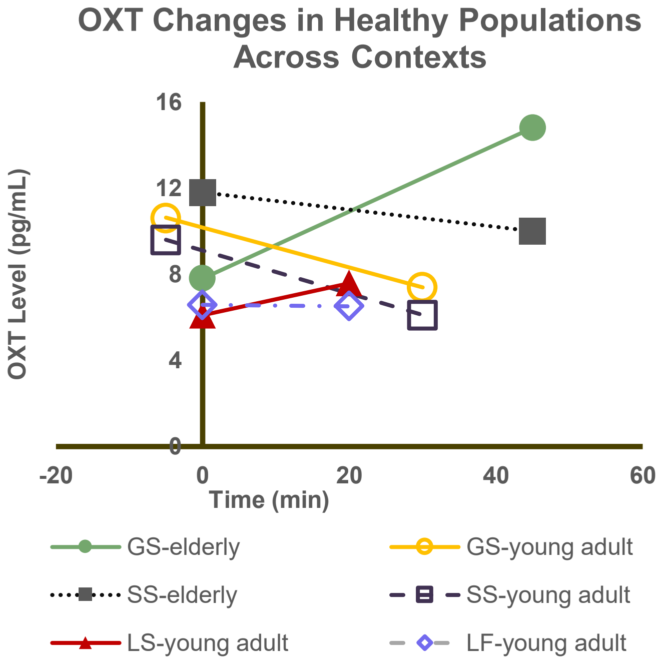

(B)

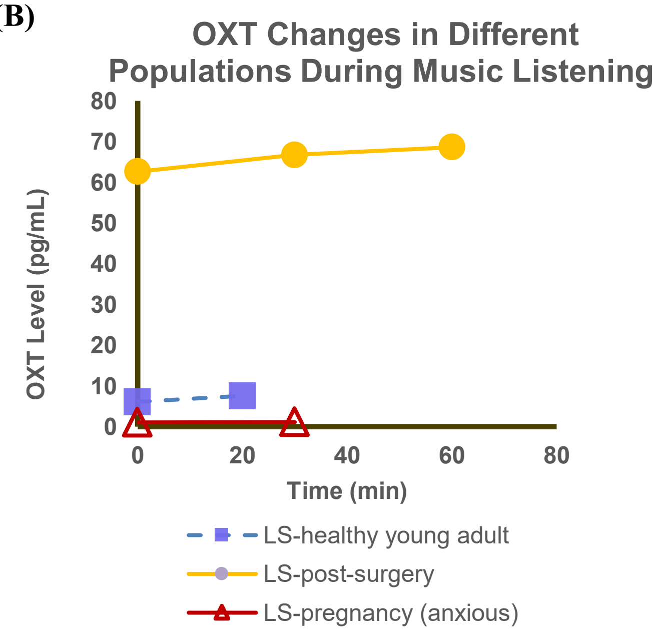

(C)

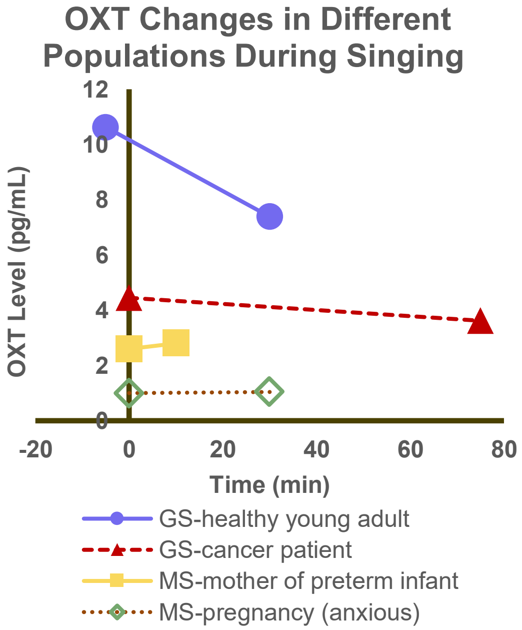

Supplementary Figure 1. Peripheral Oxytocin (OXT) Changes Induced by Music Over Time

Note. Supplementary Figure 1 was created by the first author based on the data from Bowling et al. (2022), Fancourt et al. (2016), Filippa et al. (2023), Good & Russo (2022), Nilsson (2009), Ooishi et al. (2017), and Wulff et al. (2021). (A) OXT Changes in Healthy Populations Across Contexts; (B) OXT Changes in Different Populations During Music Listening; (C) OXT Changes in Different Populations During Singing. Each data point represents the mean OXT concentration (pg/mL), corrected by sampling time. Time 0 indicates the start of the intervention. GS = Group singing; LF = Listening to fast music; LS = Listening to slow music; MS = Maternal singing; SS = Solo singing.
